# Case report: Clinical experience of treating pembrolizumab-induced systemic capillary leak syndrome (SCLS) in one patient with metastatic gastroesophageal junction squamous cell carcinoma

**DOI:** 10.3389/pore.2023.1611330

**Published:** 2023-09-08

**Authors:** Hua Ni, Xinjia Ding, Shikai Wu, Xuan Jin

**Affiliations:** Department of Medical Oncology, Peking University First Hospital, Beijing, China

**Keywords:** immune checkpoint inhibitor, pembrolizumab, adverse effect, systemic capillary leak syndrome, SCLS

## Abstract

Systemic capillary leak syndrome (SCLS) is a rare and complex adverse effect of immune checkpoint inhibitors (ICIs). The diagnosis of drug-induced SCLS is based on diffuse infusions of exudative fluid into the interstitial areas and the exclusion of other causes. The best management of ICIs-induced SCLS is not settled, though proper supportive care and corticosteroids were commonly applied as the first-line treatment. In our patient with advanced gastroesophageal junction squamous cell carcinoma, although ICIs-induced SCLS was successfully controlled with corticosteroids, the patient soon experienced cancer progress and died of pulmonary infections. Based on our experience and the reported cases by other hospitals, different stages of SCLS might respond differently to the same treatment. Therefore, a grading of ICIs-induced SCLS might help to stratify the patient for different treatment strategies. Besides, corticosteroids-sensitive patients, though waived from deadly SCLS, might be at higher risk of cancer progress and subsequent infections due to the application of corticosteroids. Considering that the inflammatory factors should be closely involved in the development of ICIs-induced SCLS, targeted therapy against the driver inflammatory cytokine might offer treatment regimens that are more effective and safer.

## Introduction

Systemic capillary leak syndrome (SCLS) is a rare adverse effect of immune checkpoint inhibitors (ICIs) characterized by the increase of capillary permeability, which allows the accumulation of fluids and protein in the interstitial or extravascular sites [1–3]. Common clinical manifestations of SCLS include diffuse pitting edema, exudative serous cavity effusions, non-cardiogenic pulmonary edema, hypotension, and SCLS might proceed into hypovolemic shock and multiple-organ failure if no proper intervention was conducted [2, 4].

The mechanism of secondary SCLS was not fully explained, but scholars suggested that inflammatory factors-related vascular endothelial dysfunction might be a main contributor [2, 4, 5]. The standard treatment of secondary SCLS was not yet settled, but supportive care and corticosteroids are commonly applied as the primary treatment [2, 4, 6, 7]. In 2021, the first case of pembrolizumab-induced SCLS was reported, and the authors presented a combination treatment with intravenous immunoglobulins (IVIG) and axitinib that successfully resolved the symptoms where the combination of tocilizumab (an IL-6 antagonist) and high-dose intravenous corticosteroids led to no responses [5]. In our case, pembrolizumab-induced SCLS in one patient with advanced squamous cell carcinoma was successfully managed with methylprednisolone and plasma infusion, but the patient died within 2 months due to cancer progression and pulmonary infection.

Due to the rarity and complexity of ICIs-induced SCLS, the proper treatment of SCLS in patients with advanced malignancy remains challenging. Therefore, more cases should be discussed for the best management of ICI-induced SCLS.

## Case report

**FIGURE 1:**
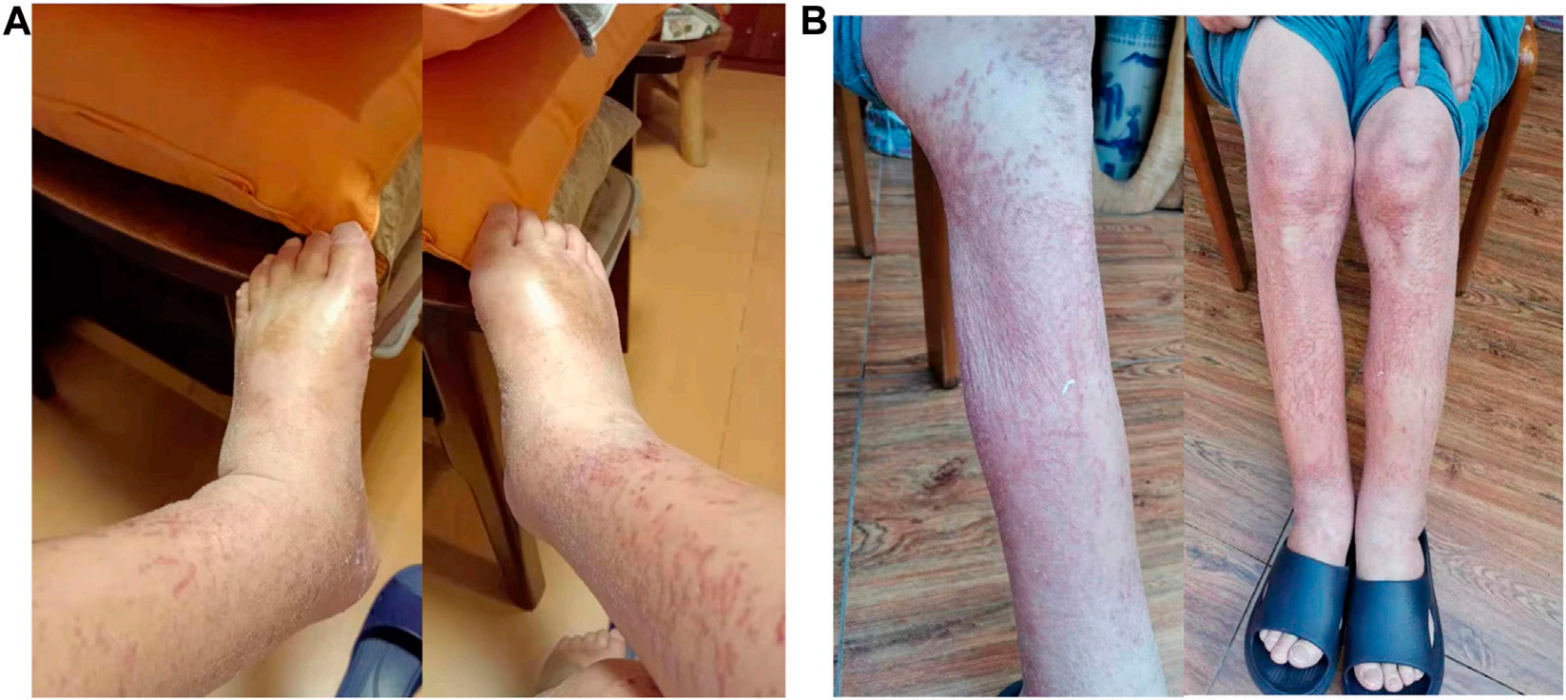
Effective management of the lower-limb edema caused by pembrolizumab-induced systemic capillary leak syndrome (SCLS) in one patient with advanced gastroesophageal junction squamous cell carcinoma with the application of methylprednisolone and plasma infusion. **(A)** Before treatment. **(B)** After treatment.

A 64 years-old male was admitted to Peking University First Hospital for new onset of dysphagia and upper abdominal discomfort. The upper gastrointestinal endoscopy revealed an ulcer-like lesion located 35–40 cm away from the incisors. The histological subtype was diagnosed as undifferentiated invasive squamous cell carcinoma. Immunohistochemical (IHC) assays revealed a CPS (combined positive score) of program death ligand-1 (PD-L1) of more than 60, proficient mismatch repair (positive MSH-2, MSH-6, PMS-2, and MLH-1 proteins), and negative human epidermal growth factor receptor 2 (HER2) expression (1+). Besides, EBV-encode small RNA *in situ* hybridization indicated an EBV-negative status. A comprehensive tumor burden evaluation was achieved through a contrast enhanced computed tomography (contrast CT) of the abdomen and pelvis, and multiple metastases to the liver, peritoneum, bone, and lymph nodes were suggested. Due to the disseminated metastases, no local treatment was performed, and the systemic treatment was started after proper nutrition supplement through gastrostomy. Paclitaxel combined with anti-programmed death 1 (PD-1) monoclonal antibody Pembrolizumab (Keyruda^®^) was administered for two cycles between 21st March and 17th April. After the first cycle of treatment, the patient’s dysphagia was significantly relieved, but he experienced moderate rashes and abdominal distention, and was admitted to the outpatient clinics for the treatment of the intolerable ascites on 13th April. Tests of the ascites indicated transudative leakage and negative tumor makers. After the second cycle of treatment, on 2nd May, the patient was admitted to the emergency department due to exacerbated rashes, severe edema in the lower limbs (Figure 1), and ascites. His blood pressure and heart rate were normal, serum albumin decreased to 29 g/L, alkaline phosphatase (ALP) increased to 216 IU/L, and γ-glutamyl transferase (GGT) increased to 135 IU/L. A diagnosis of hypoproteinemia-related edema was considered. Yet, albumin and furosemide infusions for 3 days did not alleviate the syndrome. A set of diagnostic tests was performed, but no specific reason for edema and ascites could be found: the patient had normal liver [normal alanine transaminase (ALT) and aspartate transferase (AST) level], heart [normal N-terminal pro-brain natriuretic peptide (NT-pro-BNP level)], and renal function [normal glomerular filtration rate (GFR)], normal plasma tumor markers, no fever, no endocrine dysfunctions, and no immunological disorders. Ultrasound of the lower limbs revealed no embolism but lymphedema. To be noted, the patient had a high white blood cell (WBC) count (14.6 × 10^9/L) that was marked by increased neutrophil count (12.1 × 10^9/L), monocyte count (1.04 × 10^9/L), eosinophil count (0.66 × 10^9/L), basophil count (0.08 × 10^9/L) and a decreased lymphocyte count (0.81 × 10^9/L), and high C-reaction protein (CRP) (30 mg/L) level, but no fever or elevation of procalcitonin. Based on the clinical manifestations and scarcity of causes for edema, a diagnosis of SCLS was suspected. Therefore, methylprednisolone 40 mg and plasma infusion were prescribed. Four days later, the symptoms of the patient were relieved significantly (Figure 1), and his body weight lowered from 72 to 68 kg. Besides, his WBC count lowered slightly to 12.45 × 10^9/L and was still marked by increased neutrophil count (10.55 × 10^9/L), monocyte count (0.99 × 10^9/L) and a decreased lymphocyte count (0.99 × 10^9/L). Moreover, his serum albumin level increased to 35.2 g/L. Yet, CRP, ALP and GGT levels remained high. To rule out the underlying progression of the tumor, an abdomen and pelvis CT angiography was performed shortly afterward, but no progression of the disease was noticed. The dosage of methylprednisolone was continued for 12 days and was reduced since 20th May, yet a progression of disease was noticed and the patient died shortly after on 16th July due to severe pulmonary infection.

## Discussion

Managing adverse effects is a key component of the proper application of ICIs. SCLS is a rare adverse effect of ICIs [8, 9]. To our knowledge, there have been only four reported cases of pembrolizumab-induced SCLS [5, 6, 9, 10]. The diagnosis of SCLS is based on systemic leakage of fluid into interstitials and the exclusion of other causes including sepsis, organ failure, immunological disorders, etc. [2, 11]. Due to the rarity and complexity of SCLS, the mechanism and appropriate treatment of secondary SCLS are not well explained till now.

The aberrant increase of immune cells and serum cytokines are considered as main contributors to the underlying mechanism of SCLS. Studies suggested that the inflammatory reactions evoked by cytokines including vascular endothelial growth factor (VEGF) [12], interleukin 2 (IL-2), IL-6, IL-11, IL-12, tumor necrosis factor-α (TNF-α), and interferon-γ could damage the cell junctions of the endothelial and thereby alter the capillary permeability to proteins [2, 4, 5]. In our patient, inflammatory reactions could be observed based on a high count of white blood cells and CRP levels without signs of infections or elevation of procalcitonin. But the exact driver of endothelial damage is not yet clear, and discrimination of driver cytokines and passenger cytokines (the cytokines that are triggered by the driving cytokines and process of SCLS) should be implemented to help target the true underlying cause of SCLS.

The proper treatment of SCLS is not yet settled. For idiopathic SCLS, IVIG, an immunomodulatory agent [13], was proven to be effective [14, 15]. While for drug-induced SCLS, steroids could ameliorate the symptoms significantly [2]. Besides, supportive care which includes fluid management, blood pressure control, and hemostasis supervision remains crucial, especially for patients at risk for hypovolemic shock [4, 11]. Meanwhile, patients might respond differently to the same treatment. In one case of pembrolizumab-induced SCLS reported by Mayo clinics, the patient responded poorly to steroids, and was successfully healed with IVIG and axitinib [5], a tyrosine kinase inhibitor that targets the vascular endothelial growth factor receptor 2 (VEGFR2). In our case and cases recently reported by Percik et.al [9] and Marin et.al [10], pembrolizumab-induced SCLS was successfully managed with steroids. Scholars suggested that in early SCLS patients, steroids help to alleviate the inflammatory process and thereby curb the development of SCLS; while in severe SCLS patients, the junction gap in the endothelial already formed, and steroids cannot fix the junctions and therefore cannot mitigate the symptoms [2]. Indeed, our patient had stable hemodynamics (normal blood pressure) and no signs of acute kidney injury and respond well to the steroids. But the patient reported by Mayo clinics who had unstable hemodynamics and acute kidney injuries (creatinine, 1.34 mg/dL [baseline, 0.7–0.9 mg/dL]) did not benefit from steroids. Therefore, a grading of SCLS that stratifies the patient into early, intermediate, and late phases might be useful to guide the proper treatment regimens. For the establishment of this grading system of SCLS, more evidence is warranted.

Besides, treatment against the driving cytokines might even offer us a better choice. Based on our experience, the onset of SCLS could be a sign of poor prognosis in patients with advanced cancer. In our case and the case reported by Marin et al [10], although pembrolizumab-induced SCLS was successfully curbed with steroids, the patients soon developed infections and died afterward. Apparently, SCLS could lead to a pause in anti-cancer treatment, and the application of steroids increased the risk of infections and cancer progression. Therefore, managing SCLS in cancer patients requires a better regimen that targets both the cause of SCLS and the progress of cancer and would not increase patients’ vulnerability to deadly side effects. As was introduced earlier, medications against SCLS-related cytokines should be further evaluated. In the case reported by the Mayo Clinic, tocilizumab (an IL-6 antagonist) did not raise curative effect, and bevacizumab (VEGF antagonist) induced acute deep venous thrombosis (coagulation disorder was also observed in our patient), but axitinib along with IVIG controlled the symptoms successfully and the patient survived for more than 1 year after the onset of SCLS. Therefore, medications against crucial cytokines along with IVIG might be a better choice to balance the control of both ICIs induced-SCLS and the progress of cancer.

## Conclusion

SCLS is a rare and complex adverse effect of ICIs. The inflammatory process triggered by ICIs might be the main contributor to the development of SCLS, but the exact driver cytokine of ICIs-induced SCLS is not yet clear. Steroids help to mitigate early-stage ICIs-induced SCLS, but cannot help to curb the cancer progress and might increase the risk of subsequent infections. For steroids-refractory ICIs-induced SCLS, anti-VEGF along with IVIG could be considered. Excavating the driver cytokine should lead to safer and more efficient management of ICIs-induced SCLS and thereby prolongs patients’ survival.

## Data Availability

The raw data supporting the conclusion of this article will be made available by the authors, without undue reservation.
